# A Clinical and Experimental Comparison of Time of Flight PET/MRI and PET/CT Systems

**DOI:** 10.1007/s11307-015-0826-8

**Published:** 2015-02-18

**Authors:** Daniela E. Oprea-Lager, Maqsood Yaqub, Indra C. Pieters, Rinze Reinhard, Reindert J. A. van Moorselaar, Alfons J. M. van den Eertwegh, Otto S. Hoekstra, Adriaan A. Lammertsma, Ronald Boellaard

**Affiliations:** 1 Department of Radiology and Nuclear Medicine, VU University Medical Center, PO Box 7057, 1007 MB, Amsterdam, The Netherlands; 2 Department of Urology, VU University Medical Center, PO Box 7057, 1007 MB, Amsterdam, The Netherlands; 3 Department of Medical Oncology, VU University Medical Center, PO Box 7057, 1007 MB, Amsterdam, The Netherlands

**Keywords:** PET/MRI, PET/CT, Multimodality imaging, Prostate cancer, [^18^F]fluoromethylcholine

## Abstract

**Purpose:**

The purpose of the study was to compare image quality and quantitative accuracy of positron emission tomography/magnetic resonance imaging (PET/MRI) and PET/computed tomography (PET/CT) systems with time of flight PET gantries, using phantom and clinical studies.

**Procedures:**

Identical phantom experiments were performed on both systems. Calibration, uniformity, and standardized uptake value (SUV) recovery were measured. A clinical PET/CT versus PET/MRI comparison was performed using [^18^F]fluoromethylcholine ([^18^F]FCH).

**Results:**

Calibration accuracy and image uniformity were comparable between systems. SUV recovery met EANM/EARL requirements on both scanners. Thirty-four lesions with comparable PET image quality were identified. Lesional SUVmax differences of 4 ± 26 % between PET/MRI and PET/CT data were observed (*R* ^2^ = 0.79, slope = 1.02). In healthy tissues, PET/MRI-derived SUVs were 16 ± 11 % lower than on PET/CT (*R* ^2^ = 0.98, slope = 0.86).

**Conclusion:**

PET/MRI and PET/CT showed comparable performance with respect to calibration accuracy, image uniformity, and SUV recovery. [^18^F]FCH uptake values for both healthy tissues and lesions corresponded reasonably well between MR- and CT-based systems, but only in regions free of MR-based attenuation artifacts.

**Electronic supplementary material:**

The online version of this article (doi:10.1007/s11307-015-0826-8) contains supplementary material, which is available to authorized users.

## Introduction

Multimodality imaging has improved patient care over the past decade. Non-invasive, integrated positron emission tomography/computed tomography (PET/CT) has proven to be a valuable diagnostic tool by combining *in vivo* metabolic and anatomic information [1]. Recently, several hybrid PET/magnetic resonance imaging (PET/MRI) systems have become available clinically [2]. Strengths of MRI include its high soft tissue contrast, high spatial resolution, and lack of exposure to ionizing radiation. General Electric (GE) has built a tri-modality PET/CT and MRI system (tri-modality, Discovery PET/CT 690 & 3T Discovery MRI 750) [3]. Using silicon photomultiplier tubes (SiPMTs), GE also has developed a fully integrated PET/MRI system that has time of flight (TOF) capabilities. However, this system still is a prototype and no (clinical) studies have been reported yet. Siemens has a fully integrated PET/MRI (Biograph mMR, Siemens Healthcare), which allows simultaneous PET and MRI acquisitions [4], enabling a reduction of total scan time. Philips has built a sequential PET and MRI system (Philips Ingenuity TF PET/MRI), with TOF ability [5].

Although PET/CT was rapidly incorporated into routine clinical practice, PET/MRI faces a number of technical challenges. MRI requires well-controlled magnetic and radio frequency (RF) fields [5]. However, PET photomultiplier tubes (PMTs), which are needed to convert and amplify the signal from scintillation crystals into an electronic signal [6], do not function properly in a strong magnetic field. Solutions to this have included physically separating PET and MRI units [5] or using avalanche photodiodes (APDs), which in contrast to PMTs are not affected by the magnetic field [7]. APDs, however, have poorer timing resolution than PMTs and consequently they have no TOF capability [8]. Recently, silicon PMTs (SiPMTs) have been described as a possible alternative to conventional PMTs, combining good energy and timing resolution with the ability to decode arrays of scintillator elements [9].

The second main challenge for PET/MRI is deriving accurate MR-based attenuation correction (MRAC) maps to correct corresponding PET data for tissue attenuation. Simultaneous Siemens PET/MRI systems use a four-tissue segmentation model, dividing a dedicated MR sequence in fat, air, lung, and soft tissue. Philips sequential PET/MRI systems employ a two- (air and soft tissue) or three-segmentation model (air, lung, and soft tissue). However, a limitation of all these approaches is that bone, the most attenuating tissue in the body, is not included in the segmentation process [10]. This yields underestimation of the standardized uptake values (SUVs) in bone lesions or lesions localized closed to bone structures. Furthermore, MRAC assumes uniform attenuation coefficients in lungs and suffers from MRI truncation due to the relatively small transaxial field of view (FOV; 45–50 cm) of the PET/MRI systems. All these shortcomings result in quantitative biases in the reconstructed PET images. Therefore, MRAC still requires major efforts to make PET/MRI studies accurate and robust [11].

PET/CT is used extensively in oncology for diagnosis, initial staging, restaging, therapy planning, and response monitoring of a variety of malignancies [12, 13]. Although PET/MRI is expected to be able to fulfill the same roles as PET/CT, this needs to be confirmed. There are promising initial results for several tumor types, including those seen in brain, head/neck, gynecological, and prostate [14–17]. However, just as for PET/CT, accurate SUV quantification [18] and, therefore, also accurate attenuation are required. With this in mind, it is important to know whether SUVs on PET/CT and PET/MRI are comparable.

The phantom and prospective clinical study presented here is distinct from other published work [19, 20] with respect to two aspects. First of all, it represents a particular comparison of image quality and quantitative performance of PET studies using PET/CT and PET/MR systems from the same vendor with similar hardware and software for the PET TOF gantries. Secondly, we performed PET/CT and PET/MRI studies in prostate cancer (PCa) patients using [^18^F]fluoromethylcholine ([^18^F]FCH). Its known relatively stable biodistribution, with almost no radiotracer redistribution from about 10 to 90 min post injection (p.i.) [21], enables good scan statistics. This particular setup, the use of [^18^F]FCH and the two TOF imaging systems, is suitable for evaluating the impact of MRAC on PET image quality and quantification.

## Materials and Methods

### Scanners

The study was performed using a Gemini TF-64 PET/CT system (Philips Medical Systems, Best, the Netherlands) [8] and a 3.0 Tesla Ingenuity TF PET/MRI system (Philips Medical Systems, Cleveland, Ohio, USA). Both scanners are based on similar PET hardware, with some minor modifications necessary to make it compatible with the MRI scanner (Achieva 3T X-series MRI) [5]. The PET software version on the PET/MRI is 9.7.1.0 and on the PET/CT is 9.5.1.4. Furthermore, on both scanners the same reconstruction algorithms are used, except that the attenuation correction maps (μ-maps) are different. For PET/CT, the μ-map is based on CT measurements of photon attenuation and uses a bilinear equation to convert to attenuation coefficients for 511-keV photon energies. For PET/MRI system, it is based on acquisition of a dedicated MR sequence (atMR), which subsequently is segmented into two (air and soft tissue) or three (air, lung, and soft tissue) classes of tissue, depending on the acquisition protocol (i.e., only pelvic region or body) and the ability of the software to detect lung contours [22]. The axial FOV (18 cm, with 9-cm overlap between bed positions) is the same for both systems and both have a 5.5-mm reconstructed isotropic spatial resolution.

### Phantom Measurements

Multiple phantom experiments were performed to determine specific performance characteristics of the PET/CT and PET/MRI systems, i.e., count rate linearity, calibration, uniformity, image quality, and contrast recovery. Both systems allow acquisitions in two modes: one specifically designed for the brain (brain mode, 2-mm voxels) and one for the body (body mode, 4-mm voxels). On both scanners, measurements were repeated up to five times, during a period of 1 year, using various acquisition protocols. In addition, phantom data were reconstructed using different algorithms and settings. All reconstructions included the usual corrections, such as detector normalization, decay, dead time, attenuation, as well as random and scatter corrections.

A cylindrical phantom (20-cm diameter, 9283 ml) filled with short-lived radionuclides (oxygen-15 and carbon-11) was used to assess count rate linearity over a wide range of radioactivity concentrations. The initial activity concentration was chosen to yield a single rate of 40 Mcps body dynamic acquisitions and 20 Mcps dynamic brain acquisitions. Data were reconstructed using a sinogram-based algorithm (3D-RAMLA) for the body mode and a non-sinogram-based algorithm (LOR-RAMLA) for the brain mode. Body mode images had a final voxel size of 4 × 4 × 4 mm^3^ and a spatial resolution of 5–7-mm full width at half maximum (FWHM) [5]. Brain mode images had a final voxel size of 2 × 2 × 2 mm^3^ and with similar spatial resolution. The dynamic image data was analyzed by defining one global region of interest in the phantom and calculating the average activity as a function of time. The calibration offset with regard to the true activity in the phantom was plotted versus true activity.

PET calibration and uniformity were assessed using the same cylindrical phantom as mentioned above. To assess image quality and SUV contrast recovery, the NEMA-NU2-2007 image quality (IQ) phantom (Data Spectrum Corporation, Durham, USA) was used. SUV contrast recovery was defined as the activity measured in the spheres relative to the true activity in the spheres (in %). Phantoms were filled with a 2-deoxy-2-[^18^F]-fluoro-d-glucose ([^18^F]FDG) solution using activity ranges of 1.3–3.9 (cylinder), 14.4–16.3 (spheres IQ), and 1.6–1.8 (background IQ) kBq·ml^−1^ at the scan start time. Samples were taken and measured in a gamma well counter (Wallac 1480 Wizard 3” automatic gamma counter, Perkin Elmer Life Sciences, Zaventem, Belgium) in order to accurately define the activity in the phantoms and to be able to assess the calibration offset of the two scanners. In the IQ phantom, the sphere to background radioactivity concentration ratio was set to ~10:1. Phantoms were scanned using the following acquisition protocols. First, using a single bed position, a 15-min acquisition was performed. Data were reconstructed using time of flight (blob-os-tf) [8] and sinogram-based (3D-RAMLA) algorithms, both resulting in a final voxel size of 4 × 4 × 4 mm^3^ and a spatial resolution of 5–7-mm FWHM. In clinical practice, blob-os-tf is used for static images and 3D-RAMLA for dynamic acquisitions. Second, a multi-bed position protocol, with 2 min per bed position, was performed. In addition, the cylindrical phantom was scanned at a single bed position for 15 min in (dynamic) brain mode and reconstructed using a line of response-based reconstruction algorithm (LOR-RAMLA). This resulted in a final voxel size of 2 × 2 × 2 mm^3^ and the same spatial resolution as mentioned above. For the cylindrical phantom, image data acquired with and without the use of MR coils were assessed by measuring global and local (volumes of interest (VOIs) ~8 ml) calibration offsets and by visual inspection for artifacts. Data acquired with the IQ phantom were assessed for SUV performance compliance with European Association of Nuclear Medicine/Research Ltd (EANM/EARL) specifications [23].

#### Specific Parameters and Settings for Phantom PET/MRI Studies

 The atMR sequence was used to generate μ-maps for the phantom studies. Specific attention to several points was required. Firstly, it is known that the signal from phantom walls is not detected by MRI. Therefore, attenuation by phantom walls is not corrected for, resulting in a constant underestimation of MRAC images (~5.5 % for 6-mm wall thickness). Secondly, for atMR acquisition on the MRI, phantoms were filled with a saline solution (~0.48 %) in order to approximately match MR relaxation factors with those of patients. In addition, some MR processing parameters, such as inhomogeneity corrections, needed to be adjusted. Finally, dedicated MR sequences and image processing settings based on the two-class segmentation (water and air) were used in order to obtain appropriate μ-maps for the IQ phantom.

### Patient Studies

The clinical study was approved by the Medical Ethics Review Committee of the VU University Medical Center, Amsterdam, the Netherlands. All patients participating in the study provided written informed consent for undergoing additional PET/MRI examinations, following PET/CT scans acquired for clinical purposes. Inclusion criteria comprised the following: (1) ability to remain supine for an additional 60 min in the PET/MRI scanner after undergoing the PET/CT examination and (2) clinical indication for performing an [^18^F]FCH PET/CT scan. Exclusion criteria were the standard absolute MRI contraindications (e.g., pacemaker, magnetic metal implants, neuro-stimulator, etc.). Relative (MRI) contraindications were claustrophobia and present or prior employment as a metalworker. For the latter patients, an X-ray of the orbit was performed prior to the PET/MRI in order to exclude possible metal splinters.

A total of 12 consecutive patients with histopathologically proven adenocarcinoma of the prostate were examined. Their mean age (±SD) was 66 ± 8 years. In all patients, the clinical indication for performing the PET/CT scans was restaging. This means suspected residual or recurrent disease after previous therapy, due to biochemical prostate specific antigen (PSA) relapse. A PSA relapse was defined as a serum concentration level above 0.2 ng·ml^−1^ after radical prostatectomy (RP) and more than 2 ng·ml^−1^ above the nadir value in patients treated by means of external beam radiotherapy (EBRT) [24]. All examinations were performed at the VU University Medical Center between November 2012 and February 2014.

Patient characteristics, including age, year of PCa diagnosis, type of primary treatment, PSA nadir, and PSA at the time of PET/CT and PET/MRI scans, as well as number, location, and visually assessed nature of all choline-avid lesions, are listed in Table 1.

**Table 1:** Patient and lesion characteristics

Pat.	Age (years)	Diagnosis PCa (year)	Previous therapy	PSAn (ng/ml)	PSAs (ng/nl)	Choline-avid lesions	Suspected benign		Suspected malignant	
							PET/CT	PET/MRI	PET/CT	PET/MRI
1	79	2002	EBRT	0.4	9.8	Prostate			+	+
						Th 7			+	+
						10R	+	+		
2	64	2011	EBRT	0.6	20.6	Prostate			+	+
						Sternum			+	+
						10 R	+	+		
						4 R	+	+		
						4 L	+	+		
3	59	2010	RALP EBRT	0.2	17.4	Acetabulum L			+	+
						Iliac L			+	+
						Para-aortic			+	+
						Pre-sacral			+	+
						Rib 5 R			+	+
						Th 7			+	+
						Inguinal L	+	+		
						Inguinal R	+	+		
4	73	2011	RALP	0.6	18	Pre-sacral			+	+
						Iliac R (1)			+	+
						Iliac R (2)			+	+
						Inguinal L	+	+		
5	59	2010	RALP Anti-HT	7.7	8.7	Iliac L			+	+
						Iliac R			+	+
						Para-aortic			+	+
						Inguinal L	+	+		
						Inguinal R	+	+		
6	57	2010	RALP LND	6	14					
7	51	2007	RALP	0.2	5.4	Iliac L			+	+
			EBRT							
			Anti-HT							
8	74	2007	Brachy	2.8	15.5	Prostate			+	+
9	72	2007	Brachy	1.0	6.5	Vesicle L			+	+
						Vesicle R			+	+
10	75	2009	Brachy	1.4	8.5	Prostate			+	+
			Cryo			Iliac R			+	+
11	63	2011	RALP	0.8	4.5	Pre-sacral (1)	+	+		
			Anti-HT			Pre-sacral (2)	+	+		
12	69	2010	RALP	0.2	2	10R	+	+		

*Pat*. patient, *PCa* prostate cancer, *PSAn* PSA nadir, *PSAs* PSA at the time of the PET scans, *EBRT* external beam radiation therapy, *RALP* robot-assisted laparoscopic prostatectomy, *Anti*-*HT* anti-hormonal therapy, *LND* lymph node dissection, *Brachy* brachytherapy, *Cryo* cryotherapy, *R* right, *L* left, *Vesicle* seminal vesicle

#### PET/CT Acquisition Protocol

 Patient preparation was similar to that required for [^18^F]FDG PET [22]. The standard activity of [^18^F]FCH was 4 MBq per kg body weight [21], resulting in an average (±SD) administered activity of 340 ± 57 MBq [^18^F]FCH. All patients underwent the same [^18^F]FCH PET/CT image acquisition protocol, including a low-dose CT (LD-CT) for anatomical localization and attenuation correction (AC), using a beam current of 30–50 mAs at 100 keV. Thirty minutes p.i., a whole-body (WB) PET sequence was performed from mid-thigh to the base of the skull with arms up. The acquisition time was 2 min per bed position with a standard number of nine bed positions. The total acquisition time for the WB PET/CT was, on average, 30 min.

#### PET/MRI Acquisition Protocol

Subsequently to the PET/CT scans, PET/MRI acquisitions were obtained at 90 ± 3 min p.i., without administering an additional activity of [^18^F]FCH. The protocol included a survey MRI for defining the scan trajectory, followed by an atMR sequence used for AC of the subsequent PET scan. Next, a WB PET scan was performed, on average consisting of eight bed positions, each with 3-min acquisition time. The acquisition time was increased with 50 % compared with the PET/CT scan to compensate for radioactive decay. The WB PET sequence also extended from mid-thigh to the base of the skull. Due to the small MRI gantry of the scanner, for the patient’s convenience, all scans were acquired with arms down. Different diagnostic MRI protocols were applied as the MRI was done for initial MR optimization and to evaluate MR image quality and MR system performance and robustness. The following sequences were acquired: total body: coronal T1-weighted fast spin-echo (FSE), coronal short-tau inversion recovery (STIR), and axial Dixon sequences; prostatic region: T2-weighted turbo spin-echo (TSE) in axial, sagittal, or coronal planes and axial diffusion-weighted sequences. The total acquisition time for the WB PET/MRI protocol was, on average, 67 min.

#### Reconstruction of PET/CT and PET/MRI Images

 PET/CT and PET/MRI data were both reconstructed using the (same) vendor provided time of flight reconstruction algorithm (blob-os-tf), resulting in a final voxel size of 4 × 4 × 4 mm^3^.

#### Image Analysis

 PET/CT and PET/MR images were interpreted in consensus by four experienced readers (two radiologists and two nuclear medicine physicians) who were aware of the clinical history of the patients. Visual analysis of the images was performed on a dedicated Philips workstation (Philips Fusion Viewer on Extended Brilliance™ Workspace), following the approach used in clinical practice: (1) the lesions were evaluated on a single modality (e.g., PET), (2) they were evaluated on PET/CT and PET/MRI independently, and (3) PET/CT and PET/MRI findings were compared.

Lesions were defined as choline-avid structures (diffuse or focal [^18^F]FCH uptake, exceeding background), incompatible with physiological uptake and with an anatomical substrate on MRI or CT. They were deemed benign or malignant based on the metastatic pattern of PCa [25, 26]. Choline-avid lymph nodes in the mediastinal, hilar, or inguinal region were considered reactive/benign in the absence of a pathological lesion [27, 28]. [^18^F]FCH uptake in all lesions (e.g., prostate, lymph nodes, bone) and certain normal tissues (e.g., lung, liver, and spleen) was assessed semi-quantitatively, by means of SUVs. This semi-quantitative approach was chosen to evaluate differences in [^18^F]FCH uptake between the PET/CT and PET/MR scans and to eliminate observer variability. The method consisted of collecting different types of SUVs in target lesions or tissues, as a clinically preferred alternative to the gold standard full kinetic modeling [29]. Therefore, VOIs were drawn semi-automatically on PET images, as described previously [29, 30]. Maximum and 3D peak SUV, normalized to body weight (SUVmax and SUVpeak), were obtained for all lesions. In addition, mean SUV using a 2–3-cm diameter VOI was derived for healthy tissues, with one exception. In this patient, a VOI in normal lung could not been drawn due to an incomplete scan trajectory of the thorax. In all other 11 patients, VOIs were drawn in the left lung in order to avoid the effects of scatter effect from the physiologically intense choline uptake in the liver.

### Statistical Analysis

Pearson product moment correlation coefficients (*R* ^2^) were used to test agreement between visual ratings, and mean and maximum SUVs derived from PET/MRI and PET/CT. Correlations were assessed for bias using linear regression. In addition, percentage differences between PET/CT- and PET/MRI-derived SUVmax were plotted as function of the PET/CT derived SUVmax to illustrate relative differences in quantitative findings.

## Results

### Phantom Studies

Count rate linearity in brain mode was comparable between the two scanners. Calibration offsets, ranging from 2 to 10 kBq·ml^−1^, were less than 10 %. However, the performance in body mode showed slight differences between the systems with a stable performance (calibration offsets less than 5 %) for PET/CT, whereas for PET/MRI, the calibration offsets were less than 10 % with activity concentration ranging from 2 to 20 kBq·ml^−1^ (supplementary Fig. 1). The images of the cylindrical phantom scanned on both systems showed uniform uptake and no visual artifacts.

**Fig. 1:**
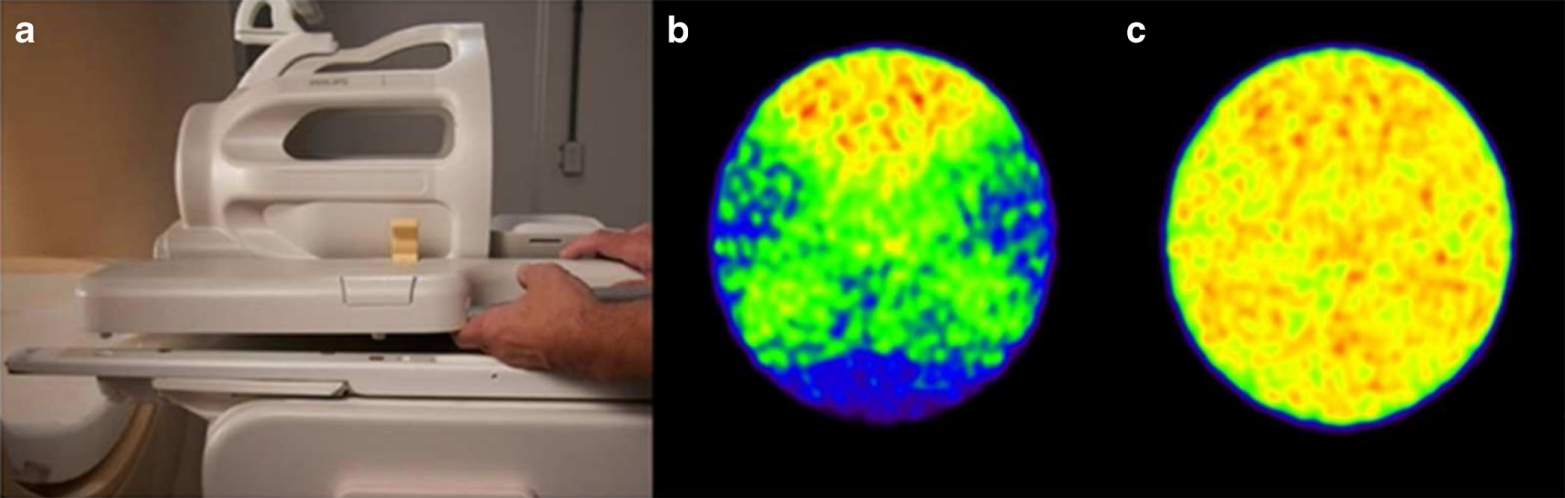
Example of coil attenuation effects: **a** image of the MR head-sense 8 coil, **b** PET acquisition including the MR head-sense 8 coil, but without a template for its attenuation correction, and **c** PET acquisition including the MR head-sense 8 coil and with a template for attenuation correction.

Global calibration accuracy and image uniformity were comparable (within ±10 %) between PET/MRI and PET/CT systems (Table 2). PET/MRI studies with additional MRI coils required the use of additional template-based AC for the coils (Fig. 1). However, template-based AC did not fully compensate for true attenuation of the coils, resulting in image artifacts in the PET images near the denser part of the coils. For example, image non-uniformities measured in a uniform source were up to 15 % when using the neurovascular coils.

**Table 2:** Calibration offsets ranges (%) for PET/MRI and PET/CT systems for different reconstruction algorithms based on five measurements within 1 year

Reconstruction	PET/MR	PET/CT
Blob-os-tf	−5.8 to −1.5	−4.3 to 2.0
3D-RAMLA	−0.6 to 4.8	−6.9 to −0.4
LOR-RAMLA	−2.1 to 3.7	−6.5 to −0.3

Calibration offset is defined as global activity of a cylindrical phantom in reconstructed PET images relative to the true activity measured using the dose calibrator

**Fig. 2:**
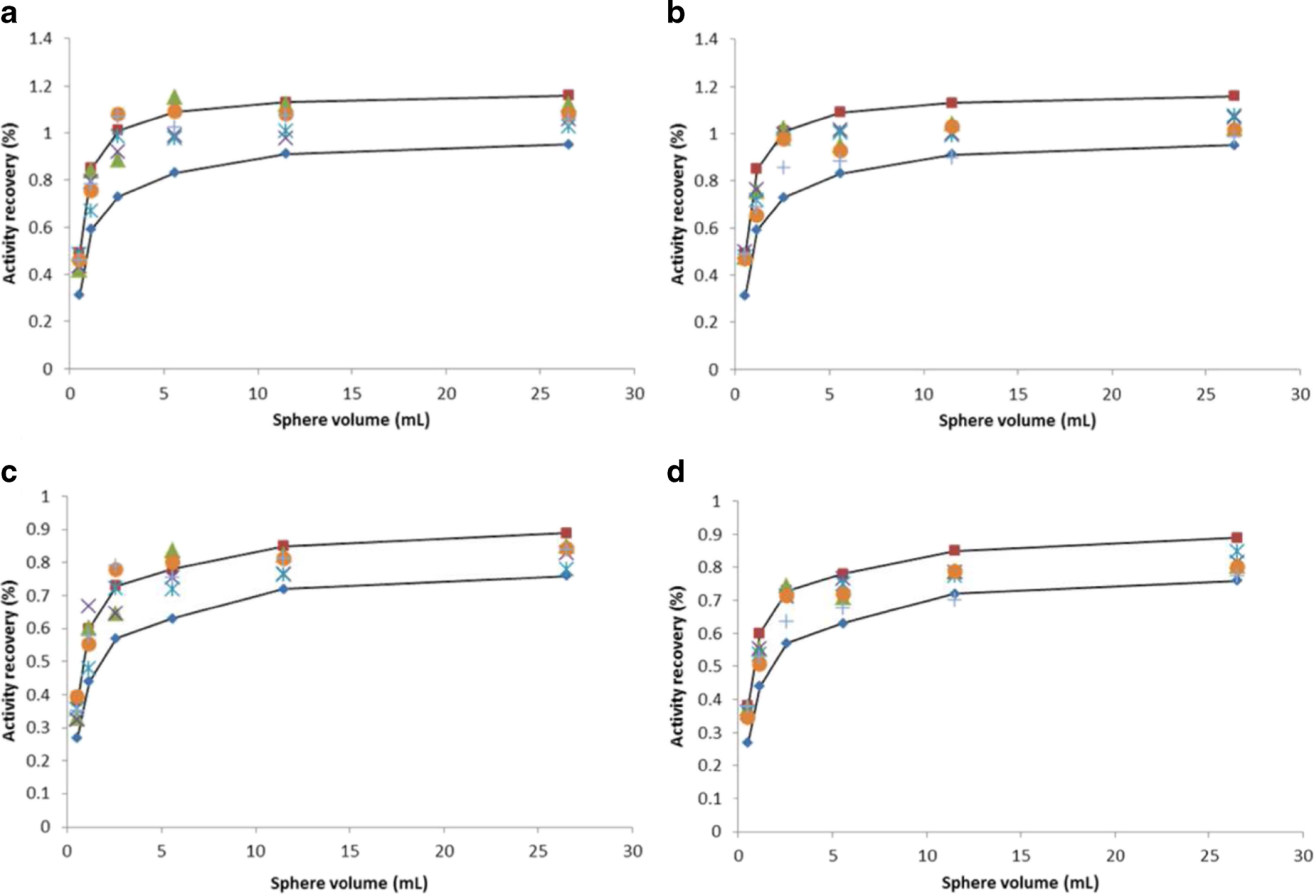
Measured activity recoveries (%) derived from the IQ phantom for both PET/MRI and PET/CT together with EARL boundaries (*solid lines*). Volumes of interest (VOIs) were defined based on a single voxel with maximum intensity (VOImax) or as an isocontour at 50 % of the maximum voxel intensity (VOIA50): **a** PET/MRI VOImax, **b** PET/CT VOImax, **c** PET/MRI VOIA50, **d** PET/CT VOIA50. Five experiments were performed during a period of 1 year, each experiment indicated by a different symbol.

The NEMA NU-2 2007 image quality phantom was measured several times on both systems The PET/CT met EANM/EARL requirements in all cases. Slightly higher contrast recoveries were found for the PET/MRI in comparison with the PET/CT (Fig. 2) for smaller spheres (<6 ml). Note that the AC by the phantom walls was not corrected because the signal from these walls (6 mm thick) is not detected by MRI. This resulted in a constant underestimation of the true recovery coefficients (5.5 % lower activity concentration).

### Patient Results

A total of 12 benign and 22 suspected malignant lesions were identified on both PET/MRI and PET/CT systems. Coexistence of benign and malignant lesions was observed in five patients. In three patients, no cause for an elevated PSA could be identified.

**Fig. 3:**
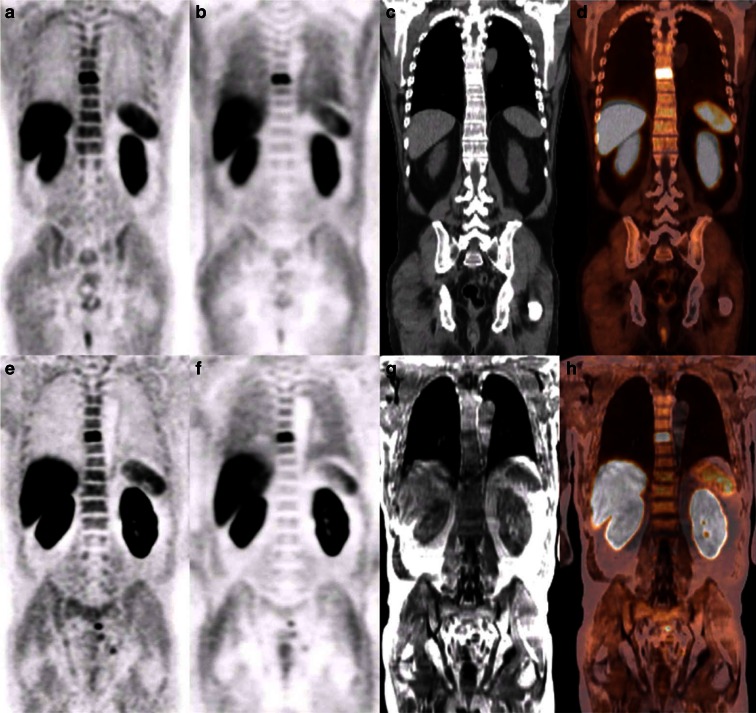
[^18^F]FCH PET/CT and PET/MRI scans of patient 3 with recurrent prostate cancer and a PSA of 17.4 ng·ml^−1^. Both scans show comparable PET image quality in a suspected bone metastasis in the seventh thoracic vertebra: **a** attenuation corrected PET image of the PET/CT, **b** non-attenuation corrected PET image of the PET/CT, **c** low-dose CT image, **d** fusion image of [^18^F]FCH PET/CT, **e** attenuation corrected PET image of the PET/MRI, **f** non-attenuation corrected PET image of the PET/MRI, **g** MR-derived attenuation image, **h** fusion image of [^18^F]FCH PET/MRI.

Benign/reactive lymph nodes were found in seven patients and were localized in the mediastinum (*n* = 2), the lung hilar region (*n* = 3), and the inguinal zone (*n* = 5). In one patient, two pre-sacral lymph nodes with slightly increased choline uptake were seen. The intensity of uptake was less than on a PET/CT scan performed previously, consistent with a response to radiotherapy. Suspected malignant lesions were identified in nine patients and were represented by lymph nodes (seven iliac, two para-aortic, two pre-sacral), bone lesions (two thoracic vertebrae, one solitary acetabulum lesion, one rib, one sternal lesion), and residual/recurrent PCa (four in the peripheral zone of the prostate and two in the seminal vesicles). Visual inspection showed comparable PET image quality for all lesions between both modalities (Fig. 3).

**Fig. 4:**
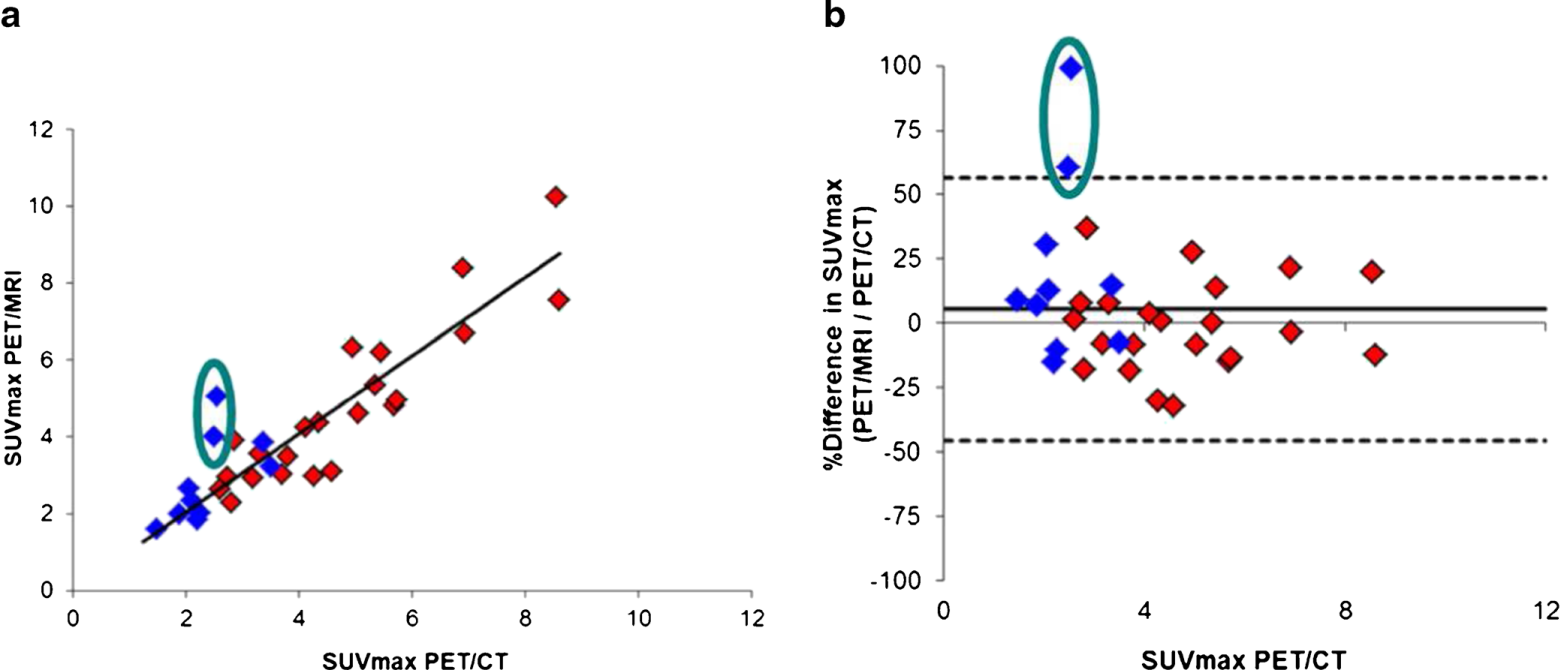
Results of the semi-quantitative analysis for benign (blue) and malignant (red) choline-avid lesions: **a** correlation between PET/MRI- and PET/CT-derived SUVmax (*R* ^2^ = 0.79, slope = 1.02), and **b** corresponding Bland-Altman plot showing large differences for two intra-thoracic lesions (*green ovals*: one para tracheal and one hilar lymph node), which were due to incorrect lung segmentation of the PET/MRI scan.

**Fig. 5:**
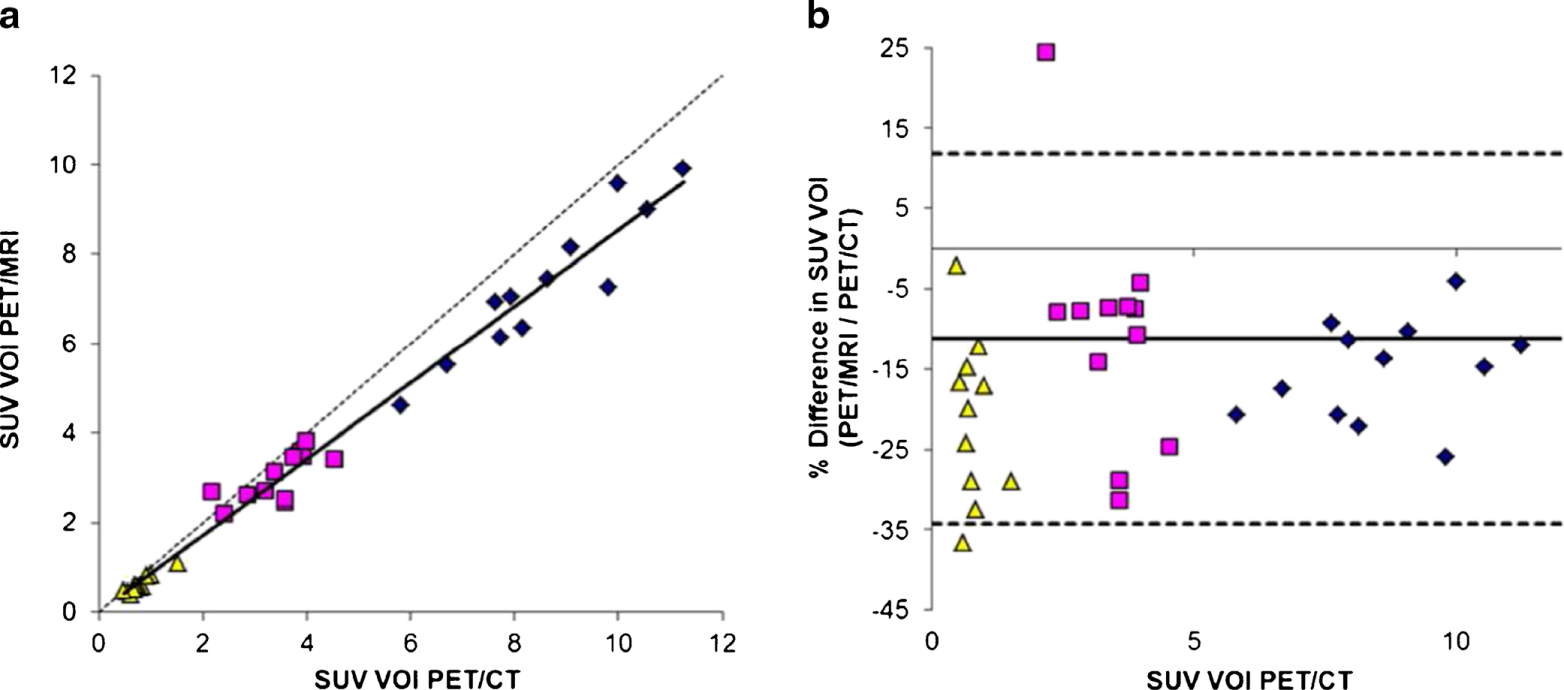
Results of the semi-quantitative analysis for healthy tissues: **a** correlation between PET/MRI- and PET/CT-derived SUVmax of liver (*blue diamonds*), spleen (*pink squares*), and lungs (*yellow triangles*) (*R* ^2^ = 0.98, slope = 0.86) together with the line of identity, and **b** corresponding Bland-Altman plot showing the largest differences in the lungs (up to −37 %).

**Fig. 6:**
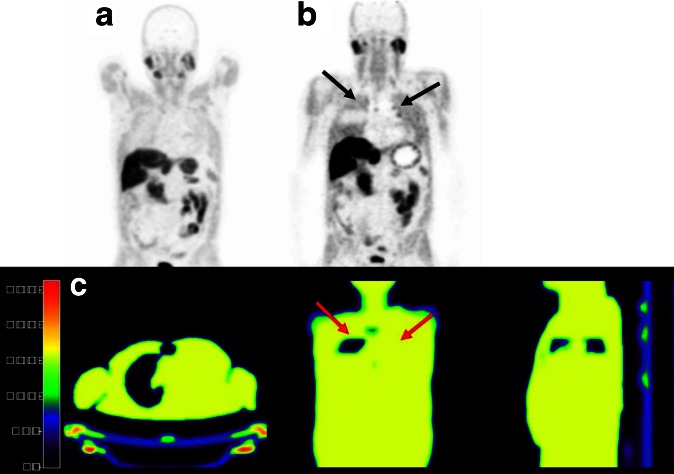
Impact of incorrect lung segmentation: **a** PET image using CT-based attenuation correction; **b** PET image using MRI-based attenuation correction, showing artificially increased uptake in the most of the lung tissue (black arrows); and **c** corresponding MRI-based attenuation map with incorrect lung segmentation (*red left arrow*: segmentation of only the apex of the right lung; *red right arrow*: no segmentation of the left lung).

The semi-quantitative lesion analysis revealed a difference of 4 ± 26 % in SUV values between PET/MRI and PET/CT data (*R* ^2^ = 0.79, slope = 1.02) (Fig. 4). In normal tissues, PET/MRI SUV values were 16 ± 11 % lower than corresponding values derived from PET/CT (*R* ^2^ = 0.98, slope = 0.86) (Fig. 5). The largest quantitative differences (up to −37 %) were found in the lungs. Two out of 12 patients showed severe artifacts in the thorax region (patient no. 1: one lymph node in the right lung hilus; patient no. 2: one mediastinal left lower para tracheal lymph node) on PET/MRI images, resulting in discrepancies of more than 50 %. These were due to incorrect lung segmentation in the MR μ-map (Fig. 6). Incorrect AC in the lungs does affect the SUV of the lesions nearby located, such as the lung hilus or mediastinal space. This is because the measured lines of response going through both the lungs and adjacently regions are not correctly adjusted for attenuation. This means that the overall attenuation along each line of response determines the amplitude of the AC. Therefore, also activity adjacent to the lungs will suffer from errors in lung segmentation.

## Discussion

The aim of this study was to directly compare image quality and quantitative performance of Philips Ingenuity TF PET/MRI with Gemini TF PET/CT systems, using phantoms and clinical data from a homogenous patient group. Because the PET gantries of both scanners are similar, it can be assumed that the performance of both PET units would be similar. To the best of our knowledge, this comparison has not previously been reported. In addition, by performing PET/CT and PET/MR studies in PCa patients with [^18^F]FCH, the relatively stable biodistribution with almost no pharmacokinetic changes between about 10 and 90 min p.i., minimizes radiotracer redistribution, and robust scan statistics are expected.

### Phantom Experiments

The phantom experiments showed comparable performances with respect to calibration accuracy, image uniformity, and contrast recovery. Due to differences between CT and MRI, some differences related to AC could be anticipated (e.g., phantom walls cannot be measured using MRI). This shortcoming, however, seemed to have a relatively low impact (not exceeding 5.5 %) on final accuracy in the phantom studies. The calibration offset of PET/MRI, together with the expected underestimation, resulted in comparable calibration accuracy for PET/MRI and PET/CT. Furthermore, a cylindrical phantom reconstructed using various available methods showed, on both scanners, uniform uptake without visual artifacts.

Some differences were seen with respect to count rate linearity, which was more accurate on PET/CT than on PET/MRI. These might be due to differences in scanner calibration software between the systems—the PET/CT is equipped with a more recent software version incorporating improved calibration and death time correction tables. Furthermore, slightly higher recoveries in the smaller spheres (<6 ml) were observed in case of the PET/MRI. These might be due to a combination of calibration offsets and shortcomings in the AC of the phantom. Apart from these minor differences, these initial phantom evaluations show that basic PET performance characteristics of PET/CT and PET/MRI systems are comparable.

MRAC remains challenging, especially when using WB PET/MRI for clinical purposes. Firstly, WB PET/MRI scan is a time-consuming procedure, requiring a stepwise optimization of the scan sequences [31]. Because of the small diameter of the MR gantry, patients are scanned with arms down. A smaller transaxial MRI field of view than the patients’ circumference may result in truncation artifacts in the MR images. Lung segmentation is another challenge, due to the diminished signal intensity of these air-containing organs in conventional MRI images. Furthermore, the current PET/MRI systems have no ability to characterize bone tissue [32], resulting in underestimation of the SUVs in bone lesions or in visceral lesions predominantly surrounded by bone structures (e.g., pelvic region).

### Clinical Evaluations

For the clinical comparison, [^18^F]FCH was used as radiotracer because of its known stable pharmacokinetic profile [24] with limited biological redistribution during the protocol period. At present, [^18^F]FCH is seen as one of the preferred tracers for restaging PCa. A systematic review and meta-analysis [33] showed a pooled sensitivity and specificity to identify all recurrent/metastatic localizations (prostatic region, bone, or lymph node) of 85.6 % (95 % CI 82.9–88.1 %) and 92.6 % (95 % CI 90.1–94.6 %), respectively.

In the present study, visual assessment of choline-avid lesions showed comparable PET image quality on both PET/CT and PET/MRI systems. Although PET/MRI showed the same detection rate of lesions compared with PET/CT, severe artifacts due to incorrect lung segmentation were identified in a small number or patients. This underlines the necessity to always inspect μ-maps, as well as attenuation- (MRAC) and non-attenuation-corrected (NAC) images to avoid incorrect conclusions from severe AC artifacts.

Similar observations with respect to lesion detection and quantitative measurements were described by Tian et al. [34] in an [^18^F]FDG PET/CT and PET/MRI comparative study of 285 patients with different malignancies. A strong diagnostic concordance between [^18^F]FDG PET/CT and PET/MRI was also found by Kohan et al. [35] in the context of lymph node staging of lung cancer patients. The authors addressed both tissue AC and anatomical nodal localization issues by using a single morphological MRI sequence (3D T1-weighted spoiled gradient echo sequence; T1w 3D GRE). Nevertheless, the applied sequence yielded a number of artifacts on the PET/MRI, limiting the confident anatomic localization in some mediastinal lesions. Drzezga and colleagues [36] described comparable reliability of data derived from [^18^F]FDG PET/MRI and PET/CT studies in patients with suspected malignant lesions. Pace et al. [37] showed an equivalent performance of the two hybrid modalities in 36 patients with breast cancer undergoing initial staging or follow-up scans.

In PCa, comparable results regarding lesion detection were described by Souvatzoglou et al. [19] in a study performed for (re)staging patients using [^11^C]choline PET/CT, followed by an integrated PET/MRI. Evaluating 36 patients with histologically proven PCa and suspected recurrent disease, Wetter et al. [20] found no difference in visual quality of PET images between [^18^F]FCH PET/MRI and PET/CT systems and concluded that the new [^18^F]FCH PET/MRI technique could be used with confidence in daily practice.

In the absence of PET/MRI artifacts (in two cases), the semi-quantitative analysis of the present data showed good correspondence in [^18^F]FCH uptake values between PET/CT and PET/MRI for both healthy tissues (*R* ^2^ = 0.98) and lesional data (*R* ^2^ = 0.79). Analysis of various lesions revealed slightly higher SUVs for PET/MRI (4 ± 26 %) than for PET/CT, while values in normal tissues were systematically lower (16 ± 11 %). The lowest SUVs were found in lungs, followed by spleen and liver. A possible reason for these differences might be a small ongoing redistribution of [^18^F]FCH with a slightly decreasing trend over time in normal or benign lesions and a stable or slightly increasing pattern in malignant lesions [28, 38, 39]. As there was a mixture of benign and malignant lesions, this could have resulted in some over- and underestimations of PET/MR versus PET/CT data. However, the observed variability is likely also related to differences in AC performance between both systems. Deriving accurate MRAC maps to correct corresponding PET data for tissue attenuation in PET/MRI remains a challenging task. Inadequate AC results in under- or overestimation of SUVs in different tissues (e.g., underestimation of the SUVs in bone lesions or lesions localized closed to bone structures, due to excluding the bone tissue in the segmentation process). In the particular case of the lungs, the theoretically assumed uniform attenuation coefficients are responsible for the lower SUVs in these organs [11]. In clinical practice, due to physiological, variable lung tissues densities, these attenuation coefficients lead to attenuation under-correction and consequently to underestimation of the SUVs in the lungs.

Higher lesional SUVs on PET/MRI were also observed by Souvatzoglou et al. [19] with good correlation between [^11^C]choline PET/MRI and PET/CT (*ρ* = 0.86). In the same study, SUVs of various normal organs, except liver, were generally lower for PET/MRI and all these differences were attributed to the uptake mechanism of [^11^C]choline. In contrast, statistically significant (*p* < 0.05) lower lesional SUV values were reported by Wetter et al. [20] for bone and prostate, with slightly higher SUVs for lymph nodes. The authors used [^18^F]choline as radiotracer for performing the scans on a simultaneous PET/MRI system. Possible explanations of the discrepancy between their findings and those from the group of Souvatzoglou were different techniques for AC and different biodistribution and biokinetics of [^18^F]FCH between early and delayed time points. The slight differences between the present findings and those of Wetter et al., while using the same radiotracer, underline the role of multiple factors contributing to underestimation in SUVs. In this respect, we agree with the German group’s hypothesis suggesting that differences in SUVs on PET/MRI are possibly related to different examination time points and MRAC.

Although in our study we focused on PET image quality and quantitative accuracy, the opportunities of assessing patients with the combined and concurrent use of PET/CT and PET/MRI will be shortly discussed. Integrated PET/CT is nowadays recognized as a preferred hybrid oncological imaging technique. It combines the anatomical information derived from CT with the functional data of tumor metabolism from PET, in an efficient whole-body setting. Nevertheless, combining PET with MRI, simultaneously or sequentially, offers new perspectives in clinical molecular imaging [40]. Three evident advantages of the new hybrid technique are the superior soft tissue contrast of MRI above CT, less radiation exposure, and the additional functional information. Nevertheless, performing PET/MRI in a whole-body setting is a time-consuming procedure, in which adequate selection of the MR sequences, clinical indications, and workflow-related aspects has to be considered.

PET/MRI is expected to be more accurate than PET/CT for tumor staging in all indications in which MRI has proven to be more valuable than CT (e.g., head and neck tumors, breast and liver malignancies, musculoskeletal neoplasms, etc.). Comparable performance is expected for lymph node staging as N-disease assessment is mainly diagnosed on functional (PET) imaging. For metastatic disease, potential advantages of PET/MRI over PET/CT depend on the site of metastatic spread [15]. In case of prostate cancer, potential PET/MRI indications are staging in patients with a positive biopsy, assessment of tumor recurrence after treatment in patients with increasing PSA, and tumor detection in case of increased PSA but negative biopsies [31].

With regard to the quantification differences between PET/CT and PET/MRI systems in our study, several shortcomings of MRAC have to be mentioned. On the one hand, there are general aspects, all vendors having difficulties with MRI truncation artifacts, not including bone structures in the segmentation process and assigning uniform lung attenuation coefficients. Specific for Philips TF PET/MRI is that it uses a three-tissue segmentation model, dividing a dedicated MR sequence in air, lung, and soft tissue. Other PET/MRI systems perform a four-segmentation model, including fat as an additional class for generating the attenuation maps. Ignoring fat may result in a small overall overestimation of the AC. Nevertheless, several approaches have been developed and evaluated to mitigate these shortcomings [41]. Furthermore, Philips designed a sequential PET/MRI system with TF ability, requiring longer acquisition time when compared with the simultaneous (e.g., Siemens) systems. However, MRAC is still work-in-progress, future clinical studies being needed to evaluate the usefulness of the different PET/MRI designs.

A limitation of the present study is represented by the sequential character of the study design, resulting in a possible redistribution of the tracer between the imaging time points of the PET/CT and PET/MRI. Nevertheless, we have specifically chosen [^18^F]FCH as radiotracer for this evaluation in order to minimize these effects. The good correspondence in SUVs across most lesions also points into this direction. Furthermore, a measurable difference in scan statistics can be expected by performing the PET/CT and the PET/MRI with a gap of 60 min. To compensate for this effect, the acquisition time on PET/MRI was increased with 50 % compared with that applied during the PET/CT scan. Another possible limitation is the variability of the used MRI protocol, as MR images were acquired in a process of optimizing imaging procedures. Yet, the same amount of lesions was identified on both PET/CT as well as PET/MRI. In a more optimal MRI setting, it is anticipated that the PET/MRI will outperform PET/CT in, e.g., differentiation of benign versus malignant lesions and identification of tumor ingrowth in surrounding soft tissue structures, including the seminal vesicles or rectal wall. However, this was not the primary aim of the present study and this remains to be studied in further studies.

## Conclusion

This study demonstrates that PET/MRI and PET/CT systems provide comparable performance with respect to calibration accuracy, image uniformity, and contrast recovery. In the absence of PET/MRI attenuation correction artifacts, there was reasonably good correspondence between [^18^F]FCH uptake in both healthy tissues and suspect lesions, yet systematically lower SUVs in normal tissues were seen for PET/MRI. Therefore, further improvement of MR-based attenuation correction is warranted. Furthermore, it is recommended to inspect attenuation corrections maps in order to avoid (quantitative) misinterpretations due to attenuation correction artifacts.

## Electronic Supplementary Material

Below is the link to the electronic supplementary material.

Supplementary Figure 1 The count rate linearity of the systems, as function of activity: **a** PET/CT and **b** PET/MRI (PDF 155 kb).
