# Supplementary material for: A Clinical and Experimental Comparison of Time of Flight PET/MRI and PET/CT Systems
Source: Mol Imaging Biol. 2015 Feb 18;17(5):714–25. doi: 10.1007/s11307-015-0826-8 (PMC4768240; doi:10.1007/s11307-015-0826-8)
Supplement: Supplementary file 1 — The count rate linearity of the systems, as function of activity: a PET/CT and b PET/MRI (PDF 155 kb). [file 11307_2015_826_MOESM1_ESM.pdf]

Supplemental Information

A Clinical and Experimental Comparison of Time of Flight PET/MRI and PET/CT Systems

Journal: Molecular Imaging and Biology

Daniela E. Oprea-Lager[1], Maqsood Yaqub[1], Indra C. Pieters[1], Rinze Reinhard[1], Reindert J. A. van Moorselaar[2], Alfons J. M. van den Eertwegh[3], Otto S. Hoekstra[1], Adriaan A. Lammertsma[1], Ronald Boellaard[1]

Departments of [1] Radiology & Nuclear Medicine, [2] Urology, [3] Medical Oncology, VU University Medical Center, PO Box 7057, 1007 MB, Amsterdam, The Netherlands

Corresponding author:
Daniela E. Oprea-Lager, MD.
Department of Radiology and Nuclear Medicine
VU University Medical Center
P.O. Box 7057
1007 MB Amsterdam
The Netherlands
E-mail: d.oprea-lager@vumc.nl
Tel: +31 20 4444366
Fax: +31 20 4443090

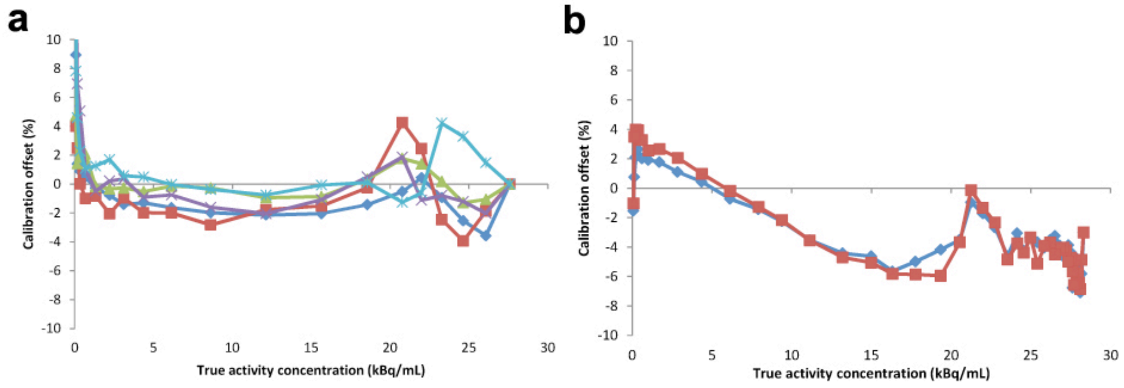

Supplementary Figure 1: The count rate linearity of the systems, as function of activity:

a PET/CT and **b** PET/MRI.
